# Supplementary material for: Prevalence of movement asymmetries in high-performing riding horses perceived as free from lameness and riders’ perception of horse sidedness
Source: PLoS One. 2024 Jul 30;19(7):e0308061. doi: 10.1371/journal.pone.0308061 (PMC11288442; doi:10.1371/journal.pone.0308061)
Supplement: S1 Text — Pdf file containing the questionnaire about rider-perceived sidedness. (PDF) [file pone.0308061.s001.pdf]

S1 Text. Sidedness questionnaire

1. Do you perceive your horse as exhibiting a sidedness? If so, please grade it.

☐ No

☐ Yes, mild

☐ Yes, moderate

☐ No perception

☐ Yes, severe

2. Do you perceive your horse as having a weaker hind limb?

☐ No ☐ Yes, right ☐ Yes, left

☐ No perception

3. If one of the hind limbs is perceived as weaker, is this noticed mostly as inside- or outside limb?

☐ Inside ☐ Outside ☐ Both, noticed equally as inside and outside limb

☐ No perception

4. What gives you the perception of one hind limb being weaker than its counterpart?

____

______________(Free text answer)____

5. Do you perceive the horse as stiffer on one side when doing lateral work (which side of the horse is perceived as most stiff)?

☐ No ☐ Yes, right side ☐ Yes, left side

☐ No perception

6. Do you perceive the horse to be more difficult to bend in the neck towards either side?

☐ No ☐ Yes, to the right ☐ Yes, to the left

☐ No perception

7. Do you perceive your horse as drifting out on the circle?

☐ No ☐ Yes, on the right circle ☐ Yes, on the left circle

☐ No perception

8. Do you perceive your horse as falling in on the circle in either direction?

☐ No ☐Yes, on the right circle ☐ Yes, on the left circle

☐ No perception

9. Do you perceive your horse as leaning more on either of the reins?

☐Leans equally ☐Right rein ☐ Left rein

☐ No perception

10. Do you perceive that your horse has more difficulty to pick-up either right or left canter?

☐ No ☐ Yes, right canter ☐ Yes, left canter

☐ No perception

11. Do you perceive that your horse has more difficulty making flying changes to either the right or left?

☐ No ☐ Yes, changes to the right ☐ Yes, changes to the left

☐ No perception

12. Does your horse drift or bulge out through the shoulder? If so, which shoulder and in what direction?

☐ Right shoulder, right track ☐ Right shoulder, left track

☐ Left shoulder, right track ☐ Left shoulder, left track

☐ No perception

13. Which leg has the horse more difficulty to yield off, leg yielding?

☐ No ☐ Right leg ☐ Left leg

☐ No perception

14. Is the travers harder in either direction?

☐ No ☐Yes, to the right ☐ Yes, to the left

15. Do you perceive your horse as having more of a struggle to do the shoulder-in in either direction?

☐ No ☐ Yes, to the right ☐ Yes, to the left

16. Do you perceive your horse as having more of a struggle to do the pirouette in either direction?

☐ No ☐ Yes, to the right ☐ Yes, to the left

17. Do you perceive your horse as more often landing in either right or left canter after a fence?

☐No preference ☐ Right canter ☐ Left canter

18. Do you perceive your horse as preferring either canter when jumping?

☐No preference ☐ Right canter ☐ Left canter

19. At what level does the horse's current rider compete/train?

Easy	☐ Jumping	☐ Dressage	☐ Eventing
Medium	☐ Jumping	☐ Dressage	☐ Eventing
Difficult	☐ Jumping	☐ Dressage	☐ Eventing

20. Do you as a rider get any recurrent comments to correct your seat?

☐ Yes, description:_____________(free text answers)________________________________

☐ No

21. Do you perceive yourself as having unequal sides with one side perceived as more difficult to ride in? (Independent on which horse you ride)

☐ No ☐ Yes, right ☐ Yes, left

22. Are you right- or left-handed?

☐ Right ☐ Left ☐ Ambidextrous
