# Supplementary material for: Prevalence of movement asymmetries in high-performing riding horses perceived as free from lameness and riders’ perception of horse sidedness
Source: PLoS One. 2024 Jul 30;19(7):e0308061. doi: 10.1371/journal.pone.0308061 (PMC11288442; doi:10.1371/journal.pone.0308061)
Supplement: S2 Text — File containing descriptive and statistical results for the subset of horses with questionnaire responses collected in conjunction with gait analysis. (PDF) [file pone.0308061.s002.pdf]

S4

Results of rider-perceived sidedness, excluding data from horses with questionnaire responses collected at a time remote from the gait analysis

This supporting information file contains descriptive and statistical results for the subset of horses with questionnaire responses collected in conjunction with gait analysis (57 horses).

Table S4.1. Questionnaire response on perceived sidedness.

Question	Pre-set answer options	Answers expressed as % of responders
**1. Do you perceive your horse as exhibiting a sidedness? If so, please grade it.**	*No*	8.8% (5 of 57)
	Mild	64.9% (37 of 57)
	Moderate	24.6% (14 of 57)
	Severe	1.8% (1 of 57)
	No perception/did not answer	0% (0 of 71)
**2. Do you perceive your horse as having a weaker hind limb?**	*No*	38.6% (22 of 57)
	Yes (left or right)	61.4% (35 of 57)
	No perception/did not answer	0% (0 of 57)
**3. Do you perceive your horse as leaning more on either of the reins?**	*None*	29.8% (17 of 57)
	Left	33.3% (19 of 57)
	Right	36.8% (21 of 57)
	No perception/did not answer	0% (0 of 57)
**4. Do you perceive your horse as drifting out on the circle?**	*None*	40.4% (23 of 57)
	Left	26.3% (15 of 57)
	Right	31.6% (18 of 57)
	No perception/did not answer	1.8% (1 of 57)
**5. Do you perceive yourself as having unequal sides with one side perceived as more difficult to ride in?**	*None*	45.6% (26 of 57)
	Left	22.8% (13 of 57)
	Right	31.6% (18 of 57)
	No perception/did not answer	0% (0 of 57)

Corresponding to Table 4 in the main article, but after excluding data from horses with questionnaire responses collected at a time remote from the gait analysis. Answers based on the number of horses included (57), for question 5 an overlap of 15 answers occurred as multiple horses had the same rider, percentage reported is based on horse and not rider.

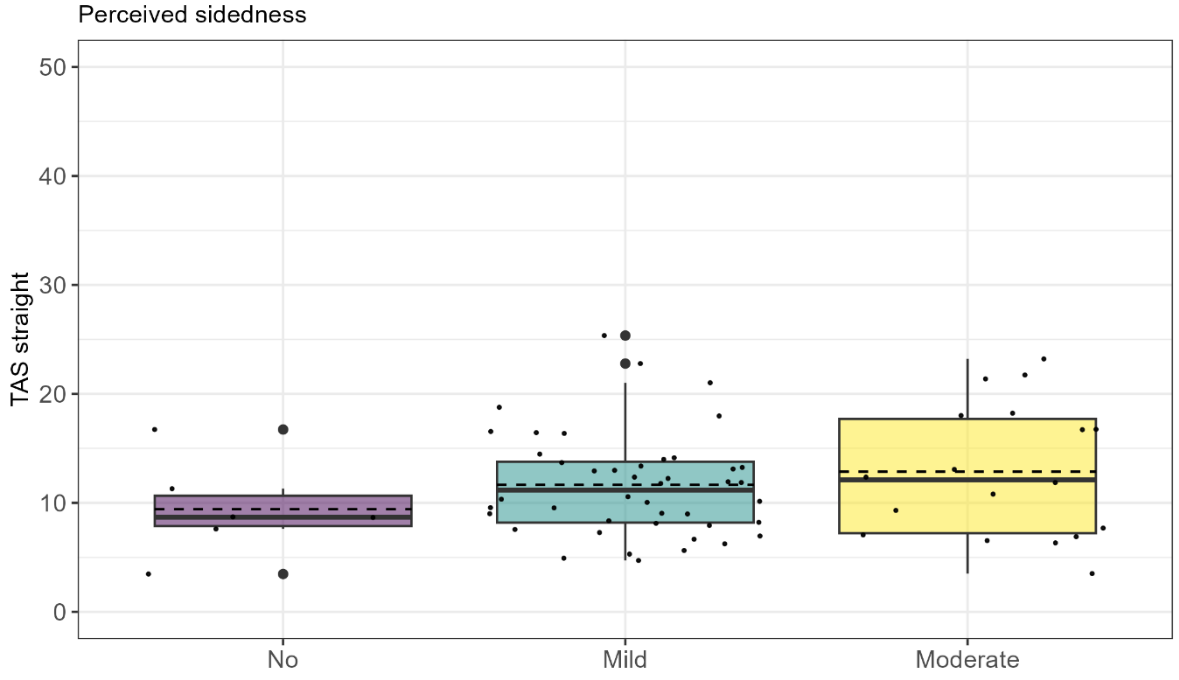

Fig S4.1. Total asymmetry score ($TAS_{straight}$) in straight-line trot plotted against level of rider-perceived sidedness. Corresponding to Fig 1 in the main article, but after excluding data from horses with questionnaire responses collected at a time remote from the gait analysis. Responses to questionnaire question 1 on riders' perception of the horse exhibiting a sidedness and degree of sidedness (no, mild, moderate) plotted against total asymmetry values obtained in straight-line trials on a hard surface. First and third quartiles represented by the upper and lower parts of the boxes. Whiskers extend to the minimum and maximum value, but no longer than 1.5 times the interquartile range. The solid line corresponds to the median value and the dashed line to the mean value. Data overlaid as small dots, outliers indicated by large dots.

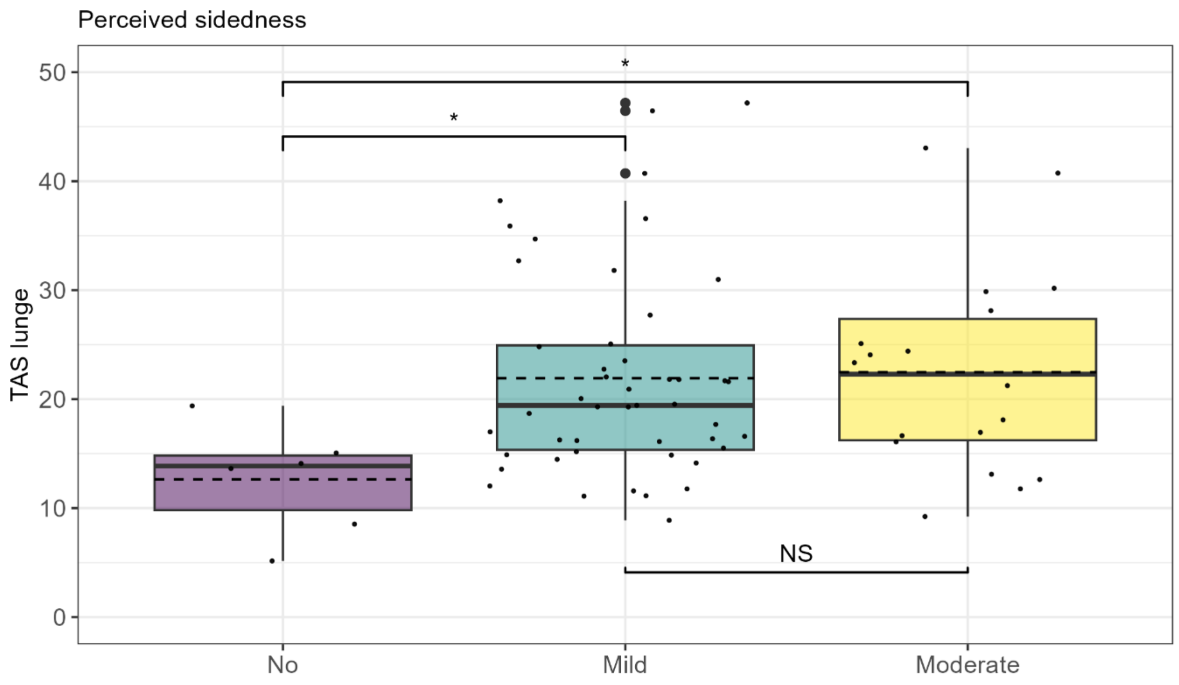

Fig S4.2. Total asymmetry score (TAS_{lunge}) from lunge trials plotted against level of rider-perceived sidedness. Corresponding to Fig 2 in the main article, but after excluding data from horses with questionnaire responses collected at a time remote from the gait analysis. Responses to questionnaire question 1 on riders' perception of the horse exhibiting a sidedness and degree of sidedness (no, mild, moderate) plotted against total asymmetry values on the lunge. First and third quartiles represented by the upper and lower parts of the boxes. Whiskers extend to the minimum and maximum value, but no longer than 1.5 times the interquartile range. The solid line corresponds to the median value and the dashed line to the mean value. Data overlaid as small dots, outliers indicated by large dots. Significant difference between groups marked with asterisk (*), no significance marked with "NS".

Table S4.2. ANOVA results for questionnaire responses tested against asymmetry parameters.

Independent variable	Dependent variable	p-value	Stride duration p-value
Sidedness (1)	$TAS_{straight}$	0.38	0.06
Sidedness (1)	TAS_{lunge}	0.02*	<0.01**
Weak hind limb (2)	PD_{min}	0.72	0.08
Weak hind limb (2)	PD_{max}	0.43	0.82

Leaning on rein (3)	HD_{min}	0.91	0.41
Leaning on rein (3)	HD_{max}	0.44	0.74
Drift on circle (4)	HD_{min}	0.65	0.40
Drift on circle (4)	HD_{max}	0.48	0.71
Drift on circle (4)	PD_{min}	0.45	0.09
Drift on circle (4)	PD_{max}	0.61	0.79
Harder side rider (5)	HD_{min}	0.34	0.44
Harder side rider (5)	HD_{max}	0.60	0.76
Harder side rider (5)	PD_{min}	0.40	0.08
Harder side rider (5)	PD_{max}	0.67	0.74

Corresponding to Table 5 in the main article, but after excluding data from horses with questionnaire responses collected at a time remote from the gait analysis Responses to questions 1-5 presented as independent variable and selected asymmetry parameters as dependent variable. Abbreviations: TAS, total asymmetry score; $HD_{min/max}$, mean difference in head minimum and maximum position between right and left strides; $PD_{min/max}$, mean difference in pelvic minimum and maximum position between right and left strides. P-values <0.05 are indicated by * and p-values <0.01 are indicated by **.
