# Supplementary material for: Prevalence of movement asymmetries in high-performing riding horses perceived as free from lameness and riders’ perception of horse sidedness
Source: PLoS One. 2024 Jul 30;19(7):e0308061. doi: 10.1371/journal.pone.0308061 (PMC11288442; doi:10.1371/journal.pone.0308061)
Supplement: S3 Text — Pdf file containing the background questionnaire. (PDF) [file pone.0308061.s003.pdf]

Name of the horse:____

Date:________________________________Location:______________________________

Background questions

Age:__________ Sex:_____________ Breed:_____________________________________

Height: __________________________ Weight: _______________________________

Total number of owners __________________ Numer of years with current owner_____________

Health

Has the horse previously been lame? YES NO

If YES:

- Which limb? □ Right front □ Left front □ Right hind □ Left hind
- When? ____
- Was a diagnosis made? If so please specify the diagnosis (e.g. hoof abscess, tendon injury).

  ____

  ____
- Was the horse treated for the lameness? If so, when?_________________________
- If the horse was treated, with what?

  ____

Is the horse regularly examined by a veterinarian with focus on the locomotion apparatus?

0 time/year 1 time/year 2 times/year >2 times/year

Is the horse's locomotion apparatus regularly treated in some other way by someone other than a veterinarian? (equitherapy, e.g. massage, acupuncture, TENS)?

____

____

Has the horse been ill in another way than lameness? YES NO

If YES, how and when? ____

____

How often is the horse receiving dental checks?

0 time/year 1 time/year 2 times/year > 2 times/year

When was the last check? _____________________________________

Were there any abnormalities found and were they treated?_____________________

What kind of bit is routinely used? ____

Is the type of biting regularly changed? YES NO
