# Supplementary material for: Prevalence of movement asymmetries in high-performing riding horses perceived as free from lameness and riders’ perception of horse sidedness
Source: PLoS One. 2024 Jul 30;19(7):e0308061. doi: 10.1371/journal.pone.0308061 (PMC11288442; doi:10.1371/journal.pone.0308061)
Supplement: Attachment — Submitted filename: Response to Reviewers.docx [file pone.0308061.s006.docx]

**Response to Reviewers PONE-D-23-43274**

Lines specified below refer to the file “Revised Manuscript with Track Changes”

**Reviewer #1**: It was a pleasure to read a well-written manuscript describing a well-designed study.

Thank you for all the valuable suggestions and feedback to improve our manuscript.

However, I would strongly encourage you to remove the ‘handedness data’ that were not acquired at the time of gait analysis. I ask clients questions on a daily basis about handedness – they struggle to remember left and right, even having ridden the horse the previous day. So even data collected at the time of examination have doubts about accuracy. Data collected at a time remote from the gait assessment cannot be considered to be accurate, particularly for riders who are riding several horses a day.
I would also strongly recommend including a statement in the limitations of the study about the potential unreliable nature of contemporaneous rider recall.

Answer: We agree that the time difference between the gait analysis and the sidedness questionire response is a limitation. We share your concern that this might influence the results and therefore performed a test where we remove these horses from the analysis. We didn´t see any changes in the results (when excluding these horses). These results have been added as a supporting file, S4. Due to the lack of difference with these horses removed, we decided to include horses with a time difference between the gait analysis and the questionnaire response.

As you point out, the recollection for most persons not that excellent. Also, the influence of the rider's own laterality and perceived position/proprioception might influence the responses in the sidedness questionnaire. It would be more accurate if the riders answered these questions during riding and also on multiple occasions. For this study setup, this was not feasible, but we still wanted to try to capture the perceived sidedness that most riders feel (and that is described) and see if there was a clear association with vertical asymmetries. From this study, we want to show that there is not a clear association at this level. However, there might be an association if you would do a more in-depth study where you would study the riders laterality, what the riders are perceiving – as the perception might not be a reflection on reality, and also over time if the perceived sidedness changes and relation to other factors (training intensity, pasture changes etc).

The supporting info and why these 20% were included are addressed in line 236-238. A sentence have also been added to address the uncertainty of the riders recall, see line 451-452.

Title – Are you differentiating between owners and riders – please clarify here & in the manuscript.

Answer: Yes, the questionnaire of sidedness was answered by the riders (not necessarily the same person as the owner). While the owner was responsible for the horse and its participation.

This is clarified in material and methods, line 110, and the title is changed

Line 79 We do not know that lameness is present in high performing equine athletes from a variety of published studies.

And we also know that judges miss lameness – also documented previously.

Horse inspections are only in hand in straight lines therefore these so-called veterinary inspections, correctly called horse inspections, can miss lameness. Moreover, the decision is made by the Ground Jury, not the attending veterinarian, and the criterion is ‘fit to compete’ not the absence of lameness. The veterinarian merely supplies advise to the Ground Jury when requested to do so. And if you ever watch the horse inspection (‘trot up’) at an international showjumping competition you will realise that is a token inspection, with the majority of horses being presented very badly, making it virtually impossible to make an accurate assessment.
Please amend the text accordingly.

Answer: As you point out, the circumstances under which the horse inspections take place are indeed suboptimal. The subjective assessments are limited and we have tried to suggest that the regular checks by experienced owners and riders and horse inspection should give a higher possibility of an abnormality being detected but as you highlight we also believe this isn´t always the case. This is in comparison to lower-level horses with e.g. hobby owners that usually are not looked at regularly by professionals or by veterinarians.

For eventing horses at a high level have a high prevalence of asymmetries been shown. There are also some studies which assess lameness subjectively in high-level horses but to our knowledge no study based on objective measurments have been done.

According to Swedish law should all horse inspections be carried out by a veterinarian and in FEI-organised competitions there should always be an official veterinarian included in the decision.

We have tried to clarify the section. See lines 81-86.

Ridden exercise - more likely to show lameness – there are several studies that have shown that the absence of lameness in straight lines does not preclude lameness either on the lunge or when ridden. It must also be borne in mind that the absence of measurable asymmetry does not preclude lameness when present bilaterally. If you investigate horses presented for evaluation of the reason for poor performance this is a not uncommon scenario which needs to be recognised.
Please amend the text accordingly.

Answer: We agree that for achieving a good assessment of a horse with poor performance problems and other indications of lameness should the evaluation be extensive, including e.g. ridden exercise. This was not in the scope of this study. Some of the horses classified symmetric in the straight line trials could have shown abnormal asymmetries on the circle indicating a bilateral asymmetry, and it would have been interesting to study how each horse looked on the circle compared to the straight lines but this was not possible for this study. And as we have not examined these horses for lameness it would be difficult to draw any conclusions from this.

We have added a section regarding the classification as “symmetric” in relation to bilateral lameness and lungeing and ridden exercise, see lines 380-382.

Line 110 & elsewhere lungeing (not lunging – this word is commonly not spelt correctly – the ‘g’ is soft in pronunciation because of the ‘e’)

Answer: thank you, this has now been corrected.

Line183 both in straight lines and on the lunge

Answer: thank you, this has now been corrected.

Line 200 '12% within a year and 8% one year after the gait analysis' Those responses should probably not be included – see above.

Answer: We thought about this and tested if the exclusion of this data had an impact on the results. As the results don´t change when these horses were removed we have chosen to keep all the horses where the rider responded to the sidedness questionnaire. We have tried to clarify this, see lines 236-238, see also the supporting file for the results presented without these 20% of horses.

Line 240 as asymmetric

Answer: Thank you, this has now been corrected.

Tables 2 & 3 I suggest it would make more biological sense if all results were presented to no more than 1 decimal place

Answer: The results have now all been rounded to one decimal place

Table 5 Do not present p-values to more than 2 decimal places

Stride duration p-value - no more than 2 decimal places

Please mention * in legend

Answer: The p-values have been rounded to 2 decimal places and a description for * has been added.

Lines 343- 345 – I would suggest that you include mention of the fact that a full clinical examination should include ridden exercise and should also include the use of a pain-analysis tool, such as the Ridden Horse Pain Ethogram. As a clinician you can only determine the influence of a gait variant on overall performance if you evaluate the horse doing what it is supposed to do. For example, a Prix St George dressage horse may be completely comfortable in working trot, but may struggle to perform tempi changes because of underlying discomfortassociated with the measured gait asymmetry. On the other hand if the asymmetry is not associated with pain the horse may be able to perform all movements easily.

The observations in your study are really important to be aware of in pre-purchase examinations – another reason why it is important to discuss the importance of ridden exercise and evaluation of behaviour potentially reflecting discomfort.

Answer: We agree that we don’t know that the presence of vertical movement asymmetries equals lameness for these horses. We have tried to clarify this, see line 372.

Line 346 ‘High-performing horses are regularly assessed for lameness by veterinarians at competitions’ – see previous comments about the role of veterinarians in the disciplines of dressage, showjumping and eventing. It is only in endurance riding that the veterinarians make decisions.
Please amend the text accordingly.

Answer: According to Swedish law (Djurskyddsförordningen 1988:539), (which is the location for this study), should all horses competing at regional competitions be checked by a veterinarian before competitions.

For competitions organized by the FEI the decision must be based on the opinion of the veterinarian delegate, and a veterinarian needs to be on the inspection panel, but as you point out has the president of the grand jury the casting vote.

We have tried to clarify the focus in these “assessments”, see lines 383-388.

Line 352 please make sure that this refers to experimentally induced lameness

Answer: this has been clarified in the text. See line 371.

Line 368 '…….indicating that the vertical asymmetries recorded did not impair their performance' - You cannot conclude that - if the horses did have pain-induced asymmetry their performance may be improved by removal of this. Diagnosis and appropriate treatment usually results in better performance - I see this regularly!

It has also been demonstrated that horses with higher Ridden Horse Pain Ethogram scores perform less well at a variety of levels and in a variety of disciplines than horses with no detectable lameness and low ethogram scores.

Answer: We agree that the phrasing was not optimal, we have worked on the discussion, see section 390-395.

Line 372 A good rider may conceal lameness by continuously making subtle adjustments or accentuate lameness by asking a horse to work harder

A limitation of the study is that horses were not assessed ridden.

Answer: We have added and rearranged in the discussion.

Line 405 among riders (between 2, among >2)

Answer: thank you, this has now been corrected.

Line 422 Please see previous comments about ridden assessment and the use of pain recognition tools, e.g., the RHpE

Answer: We agree, it would be beneficial to study these vertical movement asymmetries in combination to a full clinical examination, where for example ridden exercises are conducted and in connection to different pain assessment tools.

**Reviewer #2**: I think this is a valuable and really interesting line of enquiry. I have only minor comments and a suggestion re the discussion as follows:

Thank you for taking the time to review our manuscript.

Line 51: Please consider referring to a previous definition of lameness within the introduction. If the reader interprets 'lameness' as 'movement asymmetry' for example, line 51 where you say "it is difficult to exclude lameness as a cause of movement asymmetries in these horses" would not make sense. As I read the introduction, I had to assume your definition of lameness, was some movement abnormality resulting from pain (?). Or replace the word 'lameness' with 'pathology?' in line 51?

Answer: Thank you, we have added to the sentence to hopefully clarify this, see line 57.

Line 81: should therefore 'be more likely to be noticed'

Answer: the section have been rewritten, see section starting in line 80.

Line 91: 3 star post the classification change in 2019?

Answer:We have tried to clarify this, the requirements were set to 3* for both before and after the change but as you point out is there a lower level after 2019..

Line 97: Could the questionnaire on horse history be provided?

Answer: We have added the background questionnaire, see supporting info S5.

Line 110: Re the lunge condition - 10 m diameter? Was this standardised?

Answer: The handlers were instructed to use a 10 m circle and this was adjusted if obviously too small or big but it was not standardized to be exactly 10m using e.g. fencing. This has been rewritten.

Line 134: Consider stating here that the aim was 25 or 20 strides following removal of strides that couldn't be used. Later, when we read how the data were 'cleaned' it led me to wonder how many useable strides were left, until I realised that 25/20 was the aim POST removal of outliers and anomalous strides.

Answer: thank you, we have tried to clarify this.

Line 166 - 170: Why is HDmin and HDmax/2 but not PDmin and max?

Answer: The total range of motion of the head increases with forelimb lameness, making the asymmetries (HDmin/HDmax) approximatly double the size compared to pelvic motion asymmetry (PDmin/PDmax). Therefore head movement asymmetries were divided with two when combined with pelvic motion asymmetries to ensure equal contribution of each into the combined measure.

Line 180: should it not read 'Ime4' not Imer?

Answer: The package used in R is called lme4 whereas the function used for the data is called lmer (found in the package lme4).

Line 221: I think having 20% of riders not complete the questionnaire on day of testing is a limitation that should be pointed out. I would be interested to know if removing these riders changed the outcome of the stats.

Answer: We have added the results of the statistic tests when these horses were removed as a supporting file, see S4. We thought about this as well but as we believed that the perceived sidedness ought to be more stable, at least in older horses, we included all horses and when we testet to exclude them the results were the same.

Table 2: Minor detail but several results are reported to 1 decimal place rather than 2 (the majority). In fact I would query whether you can report to 1/100th of a mm?

Answer: we have shifted the decimal place. Note that the results are presented as mm but are corrected to the range of motion in the horse and thereby not actual mm in reality.

Line 342: why are the 6 and 12 in square brackets?

Answer: the vertical straight lined (|nr|) around 6 and 12 are to describe that the number is an absolute value, we do not take into consideration if the asymmetry is to the left or right (giving negative or positive values).

Line 360: should read 'highlights' not highlight.

Answer: thank you, this has been corrected.

Discussion - Whilst I appreciate you must present the data and provide impartial discussion, I was not clear whether you were suggesting that a large number of competition horses are actually lame, or that we need to rethink these thresholds, or that we don't yet know? If it is that we don't yet know, perhaps a more explicit statement could be added that addresses how this current work fits in to the current debate about what is a normal level of asymmetry?

Answer: We have substantially rearranged in the discussion to try to make it easier to follow the line of thought and make the discussion clearer. As you point out, this is a difficult discussion, we do not know the significance of the vertical movement asymmetries in populations of horses perceived as sound by the owners. We believe the thresholds can be of some help in the clinical situation but they do not show in black and white if the horses movement asymmetry is caused by pain or not, especially in horses with no other signs of lameness. We do however need to keep in mind that other signs might be difficult to perceive as well, as the horses are experts at hiding pain. The high prevalence of horses with big amplitude differences is concerning but we do not know the specificity of the asymmetries to pain. At this stage, we believe the best approach to the horses classified asymmetric in this population would to keep monitoring them to see if the asymmetries change and for example increase over time or with increased workload – and thereby the clinical relevance of the asymmetry.

I think what should also be acknowledged is that the horses were not tested in a ridden situation and therefore you don't know if the asymmetry seen in the unloaded condition has any relationship to asymmetries that might occur in the ridden (and warmed up) condition.

Answer: This is indeed a relevant observation and we have added this to the discussion.

Perhaps a question too far for the questionnaire (!) but it would have been interesting to know if riders perceive sidedness pre AND post warm up - perhaps part of warm up for the individual is to achieve symmetry.

Answer: As you say this was not assessed within the questionnaire used but it would definitely be interesting to look at horses before and after training, both with the gait analysis and also the perception of the ease of the movements according to the rider and the potential sidedness.

We have added to the discussion about possible improvements.
