# Supplementary material for: Prevalence of movement asymmetries in high-performing riding horses perceived as free from lameness and riders’ perception of horse sidedness
Source: PLoS One. 2024 Jul 30;19(7):e0308061. doi: 10.1371/journal.pone.0308061 (PMC11288442; doi:10.1371/journal.pone.0308061)
Supplement: Attachment — Submitted filename: Response to Reviewers.docx [file pone.0308061.s007.docx]

**Response to reviewer PONE-D-23-43274R1**

Reviewer #1: The revised version of this paper presents a more balanced picture of the study results and limitations of the study. I would suggest that it needs to be put into a broader perspective of what has been reported from other countries. I still think that there are some additional points which need addressing.

Answer: We aim to primarily compare our findings with other studies that employ the same objective methods to measure asymmetry. We have included references of asymmetries in both polo horses, riding horses and Quarter horses, which were indeed previously missing from our text.

I also think that the authors need to be rather more transparent. This is a worrying situation with such a high percentage of potentially lame horses. The group, along with other groups, have rightfully been championing the use of objective gait evaluation. But this study highlights that either our so-called cut off points for differentiating lameness and non-lame horses are highly inaccurate, or that there is a high percentage of actively competing sports horses that are lame. This needs to be addressed in a straightforward manner in my opinion.

Answer: We agree that the high percentage of asymmetries is indeed a concerning issue. Head and pelvic vertical movement asymmetry are highly sensitive indicators of forelimb and hindlimb lameness. However, the clinical specificity of these asymmetry thresholds is currently unknown. It is essential to investigate the prevalence of these asymmetries in different horse populations and to determine the specificity of these "lameness thresholds" to establish their potential use as a screening tool for lameness.

With the current knowledge, we do not believe it is possible to define cut-off values for differentiating between lame and non-lame horses. This is due to the significant overlap in absolute asymmetry values among a large proportion of horses in this study (and other studies observing asymmetries) and studies of horses with lameness confirmed through diagnostic analgesia (Maliye et al., 2015, 2016; Persson-Sjodin et al., 2023). We have in our clinical experience also observed that in some horses, small asymmetries are significant, while in others, consistent larger asymmetries do not change over time, do not increase with different training intensities, or improve with rest. The latter could potentially indicate non-pain-related asymmetries.

This study aimed to describe the prevalence of movement asymmetries and to investigate other factors associated with asymmetries, such as sidedness, in high-performing horses. It is likely that some, or even a majority, of horses with asymmetries twice the magnitude of the “threshold” used by the Lameness Locator are indeed lame and indicate a potential welfare issue. However, without systemic or local analgesic testing, we cannot confirm this definitively.

In saying that in my clinical experience there is a subset of competition horses that have low grade lameness (for which we have a diagnosis) and with careful management stay the same year in year out. There is another subset with a similar low-grade lameness (for which we have a diagnosis) in which unquestionably the horse’s performance is compromised (we know from analysis of competition results and, when they have been educated, the riders become aware of changes in ‘rideability’ – responsiveness to cues, which they might have previously ignored [ignorance is bliss]); then there is a third subset that if you evaluate them on one day they will be mildly lame but if you follow them over time the lameness gets slowly worse.
Answer: We fully agree that low-grade asymmetry or lameness can have varying implications on different horses. Given the high prevalence of asymmetry observed in our study, subjecting all horses with low-grade asymmetry to invasive diagnostic analgesia may not be the best approach, as it is invasive, painful, and carries certain risks. We believe that the key to interpreting the significance of low-grade movement asymmetry in individual horses lies in monitoring them over time. This would help us understand how they respond to rest and training, and how the lameness affects their performance

Additional specific points to be addressed:

Line 33 No clear association was found between rider-perceived sidedness and the vertical movement asymmetry values, indicating that laterality is so far not a determinant for vertical movement asymmetry. How can you conclude laterality is not a is not a determinant of vertical movement asymmetry based on no clear association between rider perceived sidedness and movement asymmetry measurements? And the mixture of the terms laterality and sidededness creates confusion.
Answer: Thank you for pointing this confusion out. We have changed the terminology to “sidedness” in both the abstract and conclusion.

Line 35 - diagnostic anaesthesia is the way with reasonable certainty to determine if there is pain causing asymmetrical movement.

Answer: We agree that conducting a full lameness exam on all horses in the study would have been highly valuable. However, due to financial limitations and ethical concerns—such as performing injections and the risk of side effects, on horses perceived as healthy by their owners—this was not feasible. With the emergence of tools like mobile apps for gait analysis, where owners can record their own horses, there is potential for more longitudinal studies on large populations of horses that may address some of our research questions in the future. Additionally, we have added “local or systemic analgesic testing” in the sentence.

Line 83-86 This sentence is not grammatically correct. Please rephrase. Moreover it has been shown that trainers are rather poor at recognising lameness, even with training – as you cite in ref 5.

Answer: The sentence has been grammatically corrected. We agree regarding the poor recognition of lameness by trainers. However, compared to horses at lower levels that probably undergo even less scrutiny (not competing or frequently observed by trainers and judges), one might expect a higher probability of detecting lameness in high-performing horses, due to the increased number of opportunities for scrutiny.

Line 95 There are 2 publications which have used objective gait analysis in addition to those in which subjective observations have been made. It is therefore not reasonable to say 'unknown'.

Answer: Thank you, this was an unfortunate choice of words, we have rewritten the sentence to be more specific.
Contino, E.; Daglish, J.; Kawcak, C. The prevalence of lameness in FEI athletes and its correlation to performance. Proc. Am. Assoc. Eq. Pract. 2023, 69, 369-370.
Scheidegger, M.D.; Gerber, V.; Dolf, G.; Burger, D.; Flammer, A.; Ramseyer, A. Quantitative Gait Analysis before and after a Cross-country Test in a Population of Elite Eventing Horses. J. Equine Vet. Sci. 2022, 117, 104077

Line 123 presumably this is 10m diameter. Please add

Answer: Thank you, this has been added to the sentence.

Line 137 I realise that this may standard practice, but it (the outliers) could be a genuine biological observation reflecting varying discomfort - in my clinical experience it is not uncommon to see horses that very in the severity of lameness shown from stride to stride - which is why it is important to watch a horse for long enough so that you are aware of any spontaneous fluctuations in gait. I think this merits mention in the Discussion.

Answer: Thank you, this is a valid point. We acknowledge that some outliers may genuinely reflect discomfort in certain individuals and emphasize the importance of observing horses over extended periods to capture the continuity of their motion. In clinical cases, individual scrutiny allows for manual inclusion or exclusion of strides based on suspected lameness. However, when measuring a large population of horses, it is crucial to establish a standardized approach to handling data. It is also important to critically assess trial quality, such as considering whether handlers are affecting the horse's head or if external factors like passing tractors are temporarily influencing measurements. These considerations should be addressed prior to conducting any analysis of the results.

Many outliers, especially those related to head movement, often correspond to horses tossing their heads rather than indicating lameness. The outlier removal process used here does not eliminate recurring strides with more extreme values. As you point out, it is important to (watch)/measure the horses for a sufficient number of strides to capture the continuity of their motion. This approach ensures that the outlier removal does not inadvertently exclude recurrent higher values, even if the severity varies among strides. The system consistently removes extreme values that are not recurring, but as you highlight, it is dependent on collecting a sufficient number of strides. Additionally, we recognize that inter-stride variability tends to decrease with higher degrees of lameness.

Our study primarily aimed to describe the study population. These points are indeed valuable but more suited for a detailed discussion on interpreting measures in clinical settings for individual horses. Such discussions delve deeper than the scope of our current study objectives.

Line 138- 140 This is obviously required!

Answer: We agree, it is important to know how the horses behaved during the data collection for the interpretation of the data.

Line 229 varied among horses

Answer: Thank you, this has now been changed.

Line 232 soft surface

Answer: Thank you, this has now been changed.

Line 238 Total population dressage n=47, 38.2%; SJ n=61, 49.6%; E n=15, 12.2%
Responders (n=71) - much higher % of event (n=15) riders (100%), & possibly dressage (n=26) riders versus SJ (n=30) - how might that have influenced the overall results?
I still question the whole value of inclusion of data regarding sidedness – which relies on rider memory – accurate description by a rider competing the questionnaire for > 1 horse - reliance on rider perceptions – the fact that so-called sidedness could be related to a variety of factors – the presence of primary equine pain, the effect of the tack fit (on both horse & rider), the effect of rider position and force distribution, variability in rider skill level (yes even at these levels of competition), the variance in ability of rider’s to describe what they have just felt (I question riders on a daily basis), and what riders assume is normal (which may not be normal)!

Answer: One reason for the lower prevalence of responses in the Show Jumping (SJ) section is that many SJ horses were measured before the implementation of the sidedness questionnaire, whereas eventing horses were measured towards the end of the study when all riders were asked to complete the questionnaire. Since only horses with completed sidedness questionnaires were modeled in relation to their asymmetries, the results should not be influenced by who filled out the questionnaire. However, a limitation arises from the smaller number of eventing horses compared to dressage and SJ horses.

We acknowledge that perceived sidedness in horses may be influenced by various factors. Nonetheless, we aimed to address the argument that asymmetries could be explained solely by a perceived sidedness. Given that these are elite riders, their perception of sidedness should be based on substantial equestrian experience. Nevertheless, we do recognize instances where different riders may perceive a horse's sidedness differently, indicating a need for further investigation in this area.

Previous studies on sidedness have shown a significantly unequal proportion of left vs. right-sidedness, whereas in our study and other population studies, the proportion of left vs. right movement asymmetries is more balanced. This observation could suggest fewer associations to sidedness in our findings.

This study represents an initial exploration of the association between vertical asymmetries and riders' perceived sidedness in high-performing horses. The study design has several areas for potential improvement, which we have attempted to discuss. While it is unfortunate that no association between rider questionnaires and asymmetry was identified, we believe that including negative results is crucial. This approach aligns with the scope of PLOS One, and we maintain that negative results contribute to advancing knowledge and avoiding wasted effort.

Line 365 While recognising that there are variations among people's ability to detect and grade lameness to put your results into more perspective I think that you should also cite other studies that have evaluated horses subjectively that were considered by riders to be working comfortably.

Answer: Since we know the agreement between visual assessment and objective methods are low we wanted to focus on studies using objective methods.

Line 384 veterinarians

Answer: Thank you, this has been changed.

Line 397 measurements

Answer: The sentence has been rewritten.

Line 453 VERY common in my experience! One of very many myths perpetuated in the equestrian world.

Line 449- 451 good point

Answer: Here, we might also need to consider the influence of time. A rider with significant motor laterality in their own body may gradually influence the horse's sides over time to mirror the rider's own sidedness.

Line 452 - particularly for those riders who filled in a questionnaire for > 1 horse

Answer: Good point, we have added this to the sentence

It might have been helpful to have had additional questions in the questionnaire - e.g., do you have symmetrical rein (or does the horse lean on one rein); does the horse feel similar in rising trot on the left and right diagonals; does the horse put you on one diagonal preferentially; if you ride on the correct diagonal in rising trot, does the horse feel different on the left and right reins?

Answer: We agree that it would have been interesting to further investigate the rider perceived sidedness with additional questions and these are excellent suggestions.

Line 475 please be consistent in the use of terminology - if you mean sidedness then say that rather than introducing laterality which creates confusion.

Answer: Thank you, we have changed to sidedness.
